# Obesity and African Americans: Physiologic and Behavioral Pathways

**DOI:** 10.1155/2013/314295

**Published:** 2013-01-27

**Authors:** Preetha Anna Abraham, Josh Ben Kazman, Stacey Anne Zeno, Patricia Anne Deuster

**Affiliations:** Department of Military and Emergency Medicine (MEM), Uniformed Services University of Health Sciences (USUHS), Room 26, Building 53, 4301 Jones Bridge Road, Bethesda, MD 20814, USA

## Abstract

Although progress has been made to understand the association between physiological and lifestyle behaviors with regard to obesity, ethnic differences in markers of obesity and pathways towards obesity remain somewhat unexplained. However, obesity remains a serious growing concern. This paper highlights ethnic differences in African Americans and Caucasians that may contribute to the higher prevalence of obesity among African Americans. Understanding ethnic differences in metabolic syndrome criteria, functioning of the hypothalamic pituitary adrenal axis, variations in glucocorticoid sensitivity and insulin resistance, and physical activity and cardiovascular fitness levels may help to inform practical clinical and public health interventions and reduce obesity disparities.

## 1. Introduction

Overweight and obesity are chronic health illnesses affecting many children and adults in the United States [1, 2]. The health consequences of overweight and obesity are enormous, particularly the risk of developing chronic diseases such as hypertension, Type 2 diabetes mellitus (T2DM), and cardiovascular disease (CVD). Obesity disproportionately affects ethnic minorities, women and individuals from lower socioeconomic groups [3, 4]. In particular, African Americans (AAs) are disproportionately affected by obesity, diabetes, hypertension, and cardiovascular disease, and it is likely that a host of factors interact in complex, and yet unexplained, ways to contribute to these health disparities. The prevalence of overweight or obesity in African women (66%) is 1.4 times that in Caucasian (CA) women (47%) [5], and African American (AA) women may be at greatest risk for the health consequences of obesity and have an almost twofold greater risk of developing diabetes and experiencing hypertension at earlier ages; they also have significantly greater abdominal fat [6] than CA women. 

The objective of the present paper is to highlight selected ethnic differences associated with obesity by focusing on factors that contribute to obesity: metabolic Syndrome (MS) indicators, regulation of the hypothalamic pituitary adrenal (HPA) axis, glucocorticoid sensitivity (GS), insulin resistance (IR), and physical activity among AAs and Caucasians (CAs).

## 2. Metabolic Syndrome

Metabolic Syndrome (MS) is a constellation of factors used globally for identifying individuals at greatest risk for developing CVD and T2DM [7]. This cluster of interrelated risk factors for CVD and T2DM [8, 9] include glucose intolerance (T2DM, impaired glucose tolerance, impaired fasting glycaemia, or insulin resistance/IR), elevated blood pressure, high triglyceride (TG) and low high-density lipoprotein cholesterol (HDL-C) levels, and excessive waist circumference (central adiposity) [8, 10]. For use as a global tool various organizations formulated simple criteria for MS diagnosis as described by Grundy et al. [11]. The National Cholesterol Education Program-Adult Treatment Panel III (NCEP-ATP III) definition of the MS is the one most often used in the United States (USA) [12].  Table 1 shows how to confirm MS by two separate definitions: the NCEP-ATP-III and the International Diabetes Federation (IDF) on the basis of a nondiabetic population [13]. However, despite the widespread use, the effectiveness of MS criteria in early detection or prediction of disease risk across ethnic groups is very much debated [14–17]. 

MS is on the rise in USA and more prevalent among Mexican Americans compared with non-Hispanic whites and blacks and among non-Hispanic white men than non-Hispanic black men [19]. The available literature strongly indicates that the criteria for MS should be modified according to racial/ethnic differences [14, 20]. For example, despite a higher prevalence and mortality from CVD, hypertension, and other related chronic diseases, AA women typically have comparable, if not lower, rates of MS than CAs [14]— but this is only because of the current screening criteria.

The blood pressure criterion is suitable for AA and CA as AA have high rates of hypertension and is usually higher than CAs [21–23]. Although this criterion may be biased for AA, no changes in cutoffs for the criteria are needed. Furthermore, fasting glucose levels appear comparable between AAs and CAs [24], indicating that glucose is not a biased criterion. However, one striking and clinically important racial difference across MS criteria relates to dyslipidemia. A large number of studies suggest that AAs, both with and without MS, have much lower rates of dyslipidemia than CAs; that is, AAs usually have significantly lower triglyceride (TG) levels and higher high-density lipoprotein (HDL-C) levels than CAs [14–17].  Table 2 shows the anthropometric and metabolic information of AA and CA participants in one study, whereas fasting morning blood glucose, HOMA-IR (Homeostasis Model Assessment: fasting glucose (mmol/L) × fasting Insulin (mU/L)/22.5), and blood pressure did not vary by ethnicity; HDL-C was significantly higher and TG was significantly lower in AAs compared to CAs [15]. Thus, two of only five/six criteria for MS are biased such that MS might not be diagnosed in AA.

Yu et al. [25] reported that the activity of lipoprotein lipase (LPL), the enzyme that clears TG-rich lipid particles from the blood, was significantly higher in AAs than CAs. They postulated that this higher LPL activity might minimize the release of free fatty acids (FFA) from peripheral adipose tissue into the circulation to result in normal TG in the presence of IR [25]. Currently, genome-wide association studies (GWASs) are being conducted to elucidate gene-gene and protein-protein interactions and how such interactions might affect levels of TG, HDL, LDL, and other lipid classes [26]. Until then, this does not change the need to develop ethnic-specific criteria.

Another criterion for MS is waist circumference (WC), which serves as a surrogate marker for abdominal obesity [27]. Abdominal obesity is likely a stronger CVD risk factor than the more commonly used measure of BMI [28]. However, studies using advanced techniques to quantify abdominal fat suggest that waist size is not an appropriate marker of abdominal obesity for AA [29, 30]. Overall body composition appears to differ between AA and CA [31], in addition to WC. The debate on whether WC cutoffs should be based on the relationship between WC and BMI or WC and visceral adipose tissue (WC-VAT) presents another challenge [32]. In particular, AAs have comparable or slightly higher WC than CAs [33, 34]. However, WC does appear to be a superior predictor of mortality risk, regardless of ethnicity [35]. Several studies [24, 36–38] have shown that WC is highly correlated with other components of MS (serum insulin, IR, TG, HDL-C, and systolic and diastolic blood pressure), although differences among racial/ethnic groups were noted. Overall, ethnic-/race-specific cutoffs for WC may be necessary for adequately assessing health risk within different ethnic/race groups.

Currently, C reactive protein (CRP), a marker of systemic inflammation, is not included in any current definition of MS. However, CRP is independently associated with the risk of myocardial infarction and cardiovascular death [39, 40]. Non-Hispanic African adolescents with MS have higher levels of high-sensitivity CRP than CA adolescents [41]. Ethnic differences in CRP have also been noted, with AA having higher median CRP levels than CA [42]. Due to its predictive value for CVD and T2DM, CRP should be considered as a criterion of MS. Overall, if MS is to function as a valid, early screener for cardiovascular disease, clearly ethnic-specific criteria are needed.

## 3. Hypothalamic Pituitary Adrenal Axis 

The hypothalamic pituitary adrenal (HPA) axis and the locus ceruleus norepinephrine (LC-NE) system represent the primary components of the stress-responsive neuroendocrine systems, and together they manage physiological adaptations to stress to maintain homeostasis [43–45]. Obesity, and abdominal obesity in particular, has been associated with several interesting HPA axis disturbances: (1) hypersecretion of cortisol and/or ACTH in response to various stimuli; (2) heightened glucocorticoid sensitivity; and (3) increased glucocorticoid resistance to negative feedback. These particular pathways influence many systemic processes relevant to health, including metabolic, cardiovascular, and blood pressure regulation, as well as immune and inflammatory function.

Studies on the HPA axis and obesity are complicated and controversial. Some studies report comparable basal cortisol levels in obese relative to normal weight individuals [46], yet others report elevated basal cortisol levels in obese individuals [47]. Additionally, evidence suggests that diurnal rhythms of cortisol are abnormal in obese individuals, with higher afternoon/evening levels and lower than normal upon a wakening, to result in a flatter slope [48]. Interestingly, overweight AAs have been reported to have significantly lower awakening cortisol levels than overweight CAs despite having similar BMI [49], and disrupted diurnal cortisol rhythms were found among AA, but not CA men and women, as indicated by both lower a wakeing and higher bedtime cortisol levels [50]. Although the precise reasons for lower a wakeing cortisol levels and flatter diurnal slopes are unclear, chronic persistent stressors and environmental disadvantages have been proposed [51, 52]. 

Many studies have linked both acute and chronic stresses to HPA axis dysregulation: repeated episodes of stress can induce acute phase responses (APR) and chronic inflammatory processes, as indicated by elevations in CRP: the end result is CVD, T2DM, MS, and/or obesity [45, 47, 53–59]. Initially the high levels or excess cortisol are associated with increased adiposity, and particularly in the visceral fat [47], but eventually, chronic stress may result in low cortisol levels due to adrenal exhaustion and/or heightened sensitivity to glucocorticoids (GC). This is an area where further research is needed.

## 4. Insulin Resistance and Glucocorticoid Sensitivity

Obesity is characterized by a spectrum of abnormal insulin secretion, insulin resistance (IR), and T2DM. One mechanism that may lead to the development of IR is heightened GC sensitivity [18]. GC, in particular cortisol, serves as a key physiologic modulator in maintaining energy balance and mobilization of energy substrate [60]. However, the magnitude of GC effects on IR and other metabolic actions is likely determined by the density and affinity of glucocorticoid receptors (GR) in various regions of the brain and peripheral tissues. GR may be under- or overexpressed, have altered binding affinities, interact with other ligands, and/or respond to other such factors, all of which may lead to a state of GC resistance or heightened GC sensitivity [59]. Importantly, Islam et al. [61] showed that obese CA and AA men and women have higher GR densities in leukocytes when compared to normal and overweight men and women and that GR density was strongly correlated with waist circumference. Increased GC action in liver and adipose tissue would likely enhance IR, impair glucose tolerance, and promote stress-induced obesity [61]. Moreover, administration of GC such as dexamethasone, and therapeutic treatment with GC, frequently impair glucose tolerance and promote IR, although the mechanism is not fully understood [61–64]. Also, persons who become glucose intolerant after treatment with GC are more likely to develop T2DM in the future [65, 66]. In addition to GC actions and GR sensitivity, abnormal regulation of the HPA axis alone contributes to IR [67, 68]. 

Several studies have demonstrated that insulin and glucose concentrations are higher in obese individuals compared to normal weight controls [69]. Relative to ethnic/racial groups, Palaniappan et al. [70] demonstrated that fasting insulin levels were significantly higher in AA compared to CA in both normal and overweight BMI categories. Also, AA women are more likely to be diagnosed with IR and T2DM compared to CA women [71, 72]. Hypersensitivity to GC may help explain the higher prevalence of IR in AA [18]. Following treatment with dexamethasone, AA maintained greater fasting IR, as determined by HOMA, and higher fasting insulin levels than CA [18]. Additionally, AA displayed a significantly higher postmeal IR than CA, as measured by insulin areas under the curve (AUC) and higher peak postprandial insulin levels under dexamethasone conditions [18].  Table 3 presents the means for fasting glucose and insulin, calculated, HOMAs and AUC for glucose and insulin following the meal [18]. Moreover, serum insulin 50 minutes after a standardized meal was significantly higher in AAs than CAs [37]. In addition to adults, studies on prepubertal children have shown that despite having similar BMI, BF%, and visceral adiposity, insulin sensitivity is 20% lower and insulin secretion is higher in Africans versus CAs [73]. Overall, this hyperinsulinemia, induced by stress or steroids, may reflect a prediabetic state, and having access to such information would be critical for preventing the continuation of the epidemic of obesity, IR, and T2DM in AA [18]. 

Of note, the TG/HDL-C ratio, which has been reported to be closely related to IR in CA individuals, is not diagnostic for IR in AAs or Africans [15, 38]. Kim Dorner et al. [15] and others [12] have shown that although more AAs are likely to be IR, a significantly lower percentage of AA met the proposed cutoffs for the TG/HDL-C. Thus, predicting IR in AA from the TG/HDL-C is inappropriate unless different cutoff criteria are established. Additionally, although WC is an outstanding predictor of IR in CA, it is not as good predictor as in AA [15]. Therefore, traditional measures of IR to identify AA at risk lack sensitivity and specificity; however, administration of GCs may be more effective in AA for uncovering predisposition for developing T2DM and possibly CVD than current measures. Clearly promoting early identification of risk and halting the prevalence of T2DM among AAs are critical, so consensus on the refinement of criteria and tools for assessment is needed.

Finally the role of psychosocial, socioeconomic, and environmental factors in the development of IR and obesity remains to be determined. Previous work has demonstrated a relationship between positive appraisal and lower HOMA-IR and the use of negative appraisal as a coping style with increased insulin AUC following a meal [24]. The impact of these factors are important but will not be discussed further here.

## 5. Physical Activity and Cardiovascular Fitness

Physical inactivity serves as a major role in the rising prevalence of obesity, although other factors such as excess energy intake also contribute [74]. In fact, lack of physical activity is a leading contributor to the rapid rise in obesity among AA and Hispanic populations, particularly among women [75], and is the fourth leading cause of death worldwide [76]. It is clear that a sedentary lifestyle contributes to CVD, hypertension, T2DM, obesity, MS, IR, and hyperlipidemia. Although the beneficial health effects of physical activity are common knowledge and widely recognized, most individuals do not achieve the recommended levels, and many report no leisure-time physical activity [77]. 

Unfortunately, AAs are less likely to be physically active than CAs, and AA women report less leisure-time activity, fewer hours spent standing and fewer flights of stairs climbed per day than CA women [78]. Resting energy expenditure and resting fat oxidation have also been shown to be depressed with obesity and may be lower in AA than in CA women, which could lead to a greater weight gain among AA than CA [46]. Interestingly, a study conducted by Lee and Arslanian showed significant difference in fat oxidation rates between African and CA girls, but not between African and CA boys in response to the multistage graded treadmill task [79]. Thus AA women may be at highest risk for reasons above and beyond their level of activity.

**Figure 1:**
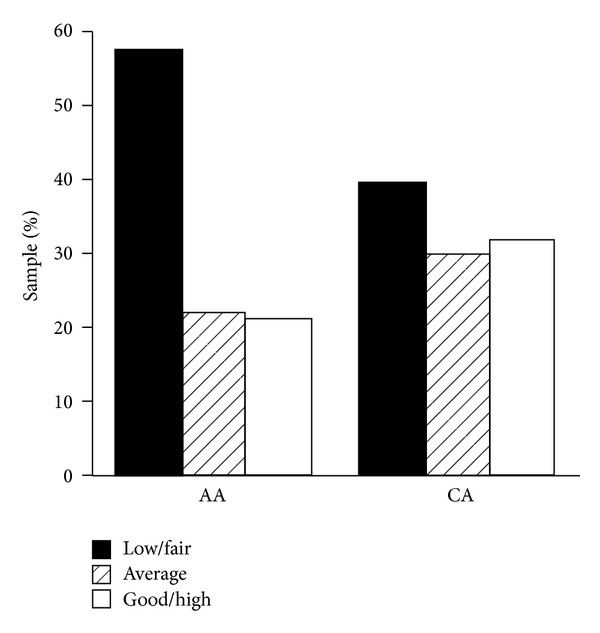
Cardiovascular fitness category by ethnicity. Abbreviations: African American (AA); Caucasian (CA). Note: Percentage of African Americans and Caucasians classified as low to fair, average, and good to high cardiovascular fitness as defined by the American College of Sports Medicine for age and gender (from [20]).

Aerobic fitness is important in the development of obesity, with a greater aerobic fitness being associated with a lower risk of obesity, MS, CVD, hypertension, and T2DM [13, 15, 20, 24, 80, 81]. AAs have a lower mean maximal aerobic exercise capacity compared to European Americans [82], and AA men have been reported to have a 7% lower exercise capacity than CA men [75]. Other cross-sectional studies have shown AA to have a lower VO_2max_ than do CA, even after adjusting for body composition [49]. Importantly, aerobic fitness is significantly and negatively related to adiposity with a high initial fitness resulting in less adipose tissue gains over a period of time [49]. Also, a strong inverse association between aerobic fitness and body fat has been reported among AAs [83]. Because of the important relation between fitness and CVD, Zeno et al. [20] examined VO_2max_, the primary index of cardiovascular fitness, in association with cardiovascular risk factors in healthy AA and CA: they found that 57% of AAs fell within the fair/low fitness group as compared to only 40% of CAs (Figure 1). Moreover, those with fair/low fitness were most likely to have multiple risk factors for CVD [20]. Finally, Gaillard et al. showed that moderate aerobic fitness is associated with reduced atherogenic lipid and lipoproteins profile in overweight or obese AA women, which could potentially lead to a lower risk of CVD [84]. Women in their very low and low aerobic fitness groups had higher glucose and insulin values, greater body weight, BMI, and %BF, and lower lean body mass when compared to the moderate fitness group [84]. Thus, it is clear that aerobic capacity or fitness is protective, and AA men and women could benefit substantially from regular exercise. Of note, many studies have also shown a strong inverse association between CRP and aerobic fitness [20, 80, 85, 86], which completes the circle: aerobic fitness, HPA axis function, and inflammation. Those who are regular exercisers and have moderate to high aerobic fitness will be less obese, have minimal systemic inflammatory processes, and have a properly functioning HPA axis. These should be the primary criteria for obesity and chronic disease risk.

Overall, educating sedentary patients to improve fitness levels by promoting regular, moderate, aerobic, or high-intensity activities will be critical for improving the health of all men and women, but in particular AA women. Although many studies are positive, the Diabetes Prevention Program Research Group demonstrated that modest aerobic exercise (brisk walking 30 min, 5 times a week) reduced the risk for developing T2DM by 58% [87]. Health care providers should inform and counsel patients to engage in regular physical activity with a goal of enhancing aerobic fitness and lowering obesity. Aerobic fitness and physical activity should always be assessed, as inactivity could be the primary criteria for early identification of those at risk for T2DM and CVD in nondiabetic, overweight, and obese populations—both in AA and CA populations.

## 6. Summary

The increasing prevalence of obesity has become a public health crisis in the United States. However, obesity disproportionately affects AA. Approximately 30% of CA adults are obese compared to 45% of AA adults [88]. Despite this trend, the most widely used treatments for overweight and obesity in U.S.A are largely ineffective [2]. The current paper focused on ethnic variations associated with MS indicators, regulation of the HPA axis, IR, GR, and physical activity—all of which are associated with obesity and chronic diseases. Ethnic-specific criteria should be established if MS is to be used as an early indicator, but factors such as aerobic fitness and CRP may be far more meaningful than conventional indicators when early intervention is the goal. The literature is clear in that a dysregulated HPA axis may lead to obesity and is a risk factor for CVD. Finally, regular physical activity is critical for overcoming obesity. AAs have lower aerobic capacity and are under greater psychosocial stress than CAs. Health care providers must asses levels of physical activity and aerobic fitness; they should encourage sedentary patients to increase physical activity regardless of their body weight and diabetic status.

## Figures and Tables

**Table 1:** Two definitions of metabolic syndrome.

Criteria	ATP III (2004)	IDF (2005)
Special instructions	Any 3 of the following 5 features:	Meet waist circumference criteria + any two of the criteria below WC
Waist circumference	Male WC ≥ 102 cm Female WC ≥ 88 cm	*Male WC ≥ 94 cm Female WC ≥ 80 cm
Blood pressure	≥130 and/or ≥85 mm Hg or on hypertension medications	≥130 and/or ≥85 mm Hg or on hypertension medications
Glucose	>5.6 mmol/L (includes diabetes)	>5.6 mmol/L (includes diabetes)
Lipids	TG > 1.7 mmol/L	TG ≥ 1.7 mmol/L or on TG Rx
	HDL-C <1.036 mmol/L for men or <1.295 mmol/L for women	HDL-C <1.036 mmol/L for men or <1.295 mmol/L for women or on HDL-C Rx

*Waist circumference (WC) based on European (Caucasian) measurements.

ATP III: adult treatment panel III; IDF: international diabetes federation; WC: waist circumference; TG: triglyceride; HDL-C: high-density lipoprotein; Rx: prescription.

From [13].

**Table 2:** Participant characteristics by ethnicity (mean ± SD).

	Caucasians (*n* = 50)	African Americans (*n* = 99)
Anthropometrics		

Height (cm)	173.3 ± 10.4	170.1 ± 10.2
Weight (kg)	83.3 ± 19.4	80.4 ± 16.8
Body mass index (kg/m^2^)	27.5 ± 05.2	27.6 ± 04.5
Waist circumference (cm)	88.1 ± 15.4	87.7 ± 12.0
Percent body fat	30.5 ± 09.6	31.9 ± 08.2

Metabolic characteristics		

Fasting glucose (mmol/L)	5.3 ± 00.7	5.1 ± 00.7
Fasting insulin (*μ*U/mL)*	10.2 ± 07.5	12.4 ± 07.8
HOMA-IR	2.4 ± 01.9	2.9 ± 02.0
Systolic BP (mm Hg)	123 ± 11.4	124 ± 13.0
Diastolic BP (mm Hg)	68 ± 08.6	69 ± 09.1
Triglycerides (mmol/L)**	1.19 ± 55.2	0.77 ± 0.49
HDL-C (mmol/L)*	1.10 ± 0.29	1.21 ± 0.32

Homeostasis model assessment of insulin resistance (HOMA-IR); blood pressure (BP); high-density lipoprotein cholesterol (HDL-C).

Note: Ethnic differences significant at *P < 0.05; **P < 0.001.

**Table 3:** Plasma glucose and insulin concentrations, calculated HOMA, and glucose and insulin AUC following a meal under placebo and dexamethasone conditions by African Americans (*n* = 63) and Caucasian Americans (*n* = 43); (mean ± SD).

	Placebo		Dexamethasone	
	African Americans	Caucasians	African Americans	Caucasians
Glucose (mmol/L)	5.3 ± 0.6	5.6 ± 0.8	6.4 ± 0.8	7.0 ± 1.2**
Insulin (*μ*IU/mL)	15 ± 11.8	11 ± 08.2**	22 ± 15.0	15 ± 09.2**
HOMA	3.7 ± 3.0	2.7 ± 2.3*	6.6 ± 05.1	4.8 ± 3.0*
Postprandial insulin AUC	7874 ± 4801	4510 ± 2878**	13715 ± 9544	7237 ± 4658**
Postprandial glucose AUC	32.0 ± 47.0	44.0 ± 49.0	72.0 ± 76	94 ± 85
Peak postprandial insulin	186 ± 127.0	107 ± 69.4**	337 ± 278	175 ± 113.7**

Homeostasis model assessment (HOMA); area under curve (AUC).

Note: *P < 0.05; **P < 0.01; *P* values represent comparisons between African Americans and Caucasians, and not treatment conditions.

From [18].
